# The Protein Phosphatase PP2A Plays Multiple Roles in Plant Development by Regulation of Vesicle Traffic—Facts and Questions

**DOI:** 10.3390/ijms22020975

**Published:** 2021-01-19

**Authors:** Csaba Máthé, Márta M-Hamvas, Csongor Freytag, Tamás Garda

**Affiliations:** Department of Botany, Faculty of Science and Technology, University of Debrecen, H-4032 Debrecen, Hungary; hamvas.marta@science.unideb.hu (M.M.-H.); fcsongor@me.com (C.F.); gtamas0516@gmail.com (T.G.)

**Keywords:** protein phosphatase PP2A, vesicle traffic, phragmoplast, cell plate, autophagy, PIN auxin efflux carriers, transcytosis

## Abstract

The protein phosphatase PP2A is essential for the control of integrated eukaryotic cell functioning. Several cellular and developmental events, e.g., plant growth regulator (PGR) mediated signaling pathways are regulated by reversible phosphorylation of vesicle traffic proteins. Reviewing present knowledge on the relevant role of PP2A is timely. We discuss three aspects: (1) PP2A regulates microtubule-mediated vesicle delivery during cell plate assembly. PP2A dephosphorylates members of the microtubule associated protein family MAP65, promoting their binding to microtubules. Regulation of phosphatase activity leads to changes in microtubule organization, which affects vesicle traffic towards cell plate and vesicle fusion to build the new cell wall between dividing cells. (2) PP2A-mediated inhibition of target of rapamycin complex (TORC) dependent signaling pathways contributes to autophagy and this has possible connections to the brassinosteroid signaling pathway. (3) Transcytosis of vesicles transporting PIN auxin efflux carriers. PP2A regulates vesicle localization and recycling of PINs related to GNOM (a GTP–GDP exchange factor) mediated pathways. The proper intracellular traffic of PINs is essential for auxin distribution in the plant body, thus in whole plant development. Overall, PP2A has essential roles in membrane interactions of plant cell and it is crucial for plant development and stress responses.

## 1. Introduction

Protein kinases and phosphatases are working as “Yin and Yang” in the phosphoregulation of proteins since their phosphorylation is mostly reversible [1]. PP2A belongs to the PPP (phosphoprotein phosphatase) family of non-metal dependent serine-threonine protein phosphatases. They are universal in eukaryotes and in most cells PP2A is the most abundant protein phosphatase [2,3]. The protein complex (the holoenzyme) is a heterotrimer: “A” subunit is a scaffold and “B” subunit is regulatory—both of these subunits (especially “B”, with multiple subfamilies) have multiple isoforms with tissue and cell compartment dependent expression patterns. The wide variability of these subunit isoforms is responsible for the large variability of localization and substrate usage of the holoenzyme. “C” is the catalytic subunit with a much lesser number of isoforms [4]. For example, *Arabidopsis* has only five PP2A/C variants, in spite of thousands of substrates and localizations of the holoenzyme [4,5]. PP2A/C implicitly catalyzes dephosphorylation of proteins at phosphoserine and -threonine amino acid side chains.

PP2A is involved in the regulation of many crucial cellular events. The functionality of a significant number of proteins—including enzymes, key players of metabolic pathways—depends on their phosphorylation state. There are two main functions of PP2A [1,2]: (i) the regulation of vital metabolic pathways and (ii) several key signal transduction pathways are characterized by cascade events with proteins undergoing reversible phosphorylation. PP2A, although not as the single protein phosphatase, has a role in this. Therefore, it is crucial for regulation of developmental events including embryo development and organogenesis. (iii) Cell cycle regulation is also implying reversible phosphorylation of proteins at regulation checkpoints. Serine-threonine phosphatases including PP2A are very important in the activation, in other cases, inactivation of these regulators. All these facts show this protein phosphatase is one of the biochemical factors that integrate the many simultaneous events in a eukaryotic cell. Thus, one could predict it is involved in the interactions between membrane compartments and vesicle traffic. As an example, PP2A regulates vesicle sorting triggered by G-protein coupled receptors [6].

The plant cell has many particular features, several of them requiring collaborations between different membrane compartments, implicitly, vesicle traffic [7]. In this review we focus on the regulation of these interactions by protein dephosphorylation. For plants, there is still a limited knowledge on the regulation of vesicle traffic by PP2A. We intend to contribute to a better understanding of this topic, raise relevant questions and suggest future research topics. We will concentrate on processes with the most relevant knowledge.

As in all eukaryotes, the microtubule (MT) cytoskeleton is crucial for vesicle traffic. A featured event is vesicle delivery during the telophase and cytokinesis of mitosis via a plant specific structure, the phragmoplast. This event is important for the assembly of cell plate, the future cell wall between daughter cells [8]. Proper assembly of the cell plate in the correct plane is crucial for normal cell patterning during plant organ development [8]. Regulation of MT assembly is achieved among others by reversible phosphorylation of microtubule-associated proteins (MAPs) (see [9] for an example). The question is: how PP2A regulates vesicle traffic during cell plate assembly (see Section 2.1)?

Autophagy is the self-destruction of cells as a response to biotic and abiotic stresses. It is triggered by blocking the target of rapamycin complex (TORC). This is a common feature for all eukaryotic cells and the TORC complex is regulated by PP2A [10]. In plants, autophagy is important not only for stress responses. Several cell types, e.g., tracheary elements, xylem fibers and cereal aleurone cells undergo programmed cell death (PCD) at maturity and autophagic processes are involved in this. Naturally senescing tissues are also characterized by autophagy [11]. The question is: how blocking of the TORC pathway and triggering of autophagy—including vesicle delivery to the vacuole—is regulated by PP2A in plants (see Section 2.2)?

The PIN family of auxin efflux carriers is a key player in the proper tissue distribution of this important plant growth regulator (PGR). PIN functioning depends on their plasma membrane distribution and intracellular recycling regulated by reversible phosphorylation (see [12] for an example). The questions are: how PP2A regulates PIN localization and functioning? How does it contribute to differential membrane localizations of different PINs (see Section 2.3)?

This review shows diverse, seemingly not interrelated pathways of plant cell vesicle traffic with the regulatory roles of PP2A as the main common point. One of the main questions is: does PP2A regulate events of endo- or transcytosis at the ER–Golgi interface? Does it influence events downstream of the ER–Golgi system? Related to the site of PP2A regulation, is there a common feature of the pathways presented in this work? There are a few mechanisms already known for PP2A-mediated regulation of plant vesicle traffic. On the other hand, we intend to emphasize the many exciting future research directions in the field.

## 2. PP2A and Vesicle Traffic in Plant Cells

### 2.1. Phragmoplast, Vesicle Traffic and Cell Plate Assembly. Involvement of PP2A in a Developmental Context

**Figure 1:**
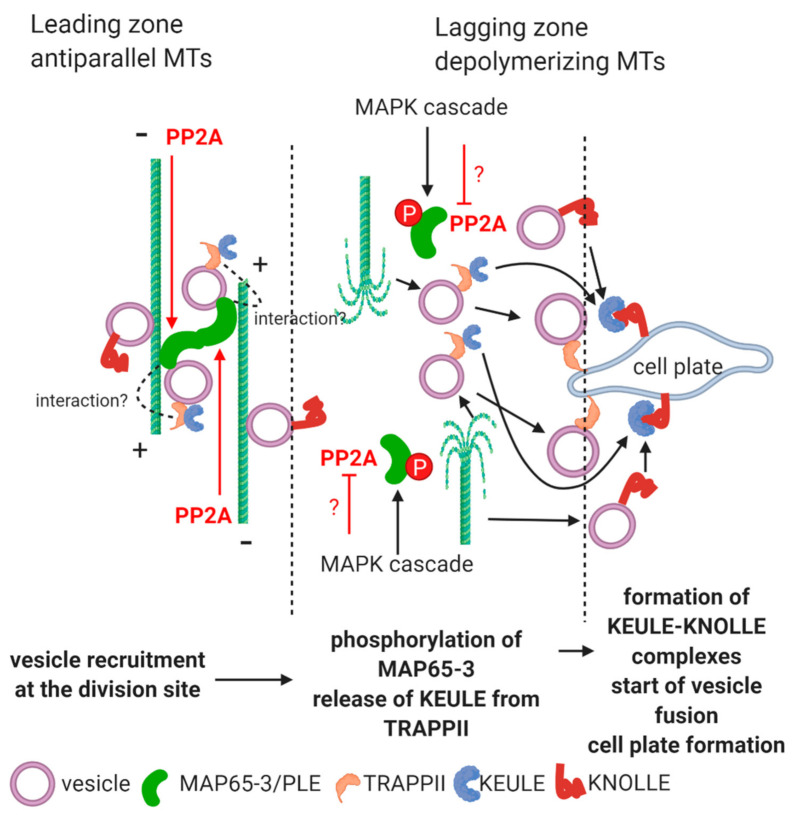
Confirmed and proposed roles of PP2A in vesicle traffic at the formation of the cell plate. For simplifying reasons, the kinesin motors that bind and deliver vesicles along phragmoplast MTs are not shown here. In the leading zone of phragmoplast, MAPK cascade-mediated phosphorylation of MAP65-3/PLE is not functional, while presumably a PP2A complex is dephosphorylating this MAP. As a consequence, it can cross-link antiparallel MTs, while it binds TRAPPII-containing vesicles that in turn, assure the KEULE-MT connection. MAP65-3 is phosphorylated via a MAPK cascade at the lagging zone, while PP2A is possibly inhibited. Thus, MAP65-3 detaches from MTs that will be destabilized as a consequence. This will release TRAPPII/KEULE containing vesicles to the site of cell plate formation and KEULE can interact with KNOLLE, allowing the t-SNARE function of the latter. This triggers vesicle fusion. Confirmed or possible pathways involving PP2A are shown in red. Question marks indicate probable, yet hypothetical events. Figure was constructed with the aid of BioRender.com.

Cell plate formation during plant cytokinesis is essential for proper mitotic division of plant cells. The formation of a new cell wall in the correct plane is important when cells are in a tissue context [8]. The two key events during cell plate formation are (i) the movement of Golgi-vesicles driven by microtubule (MT) associated motor proteins and other microtubule associated proteins (MAPs) and (ii) vesicle fusion to build up the cell plate. Both of these important events need integration of MT and endomembrane dynamics. In the following sections we summarized current knowledge and possible future research directions regarding regulation of these events by PP2A in relation to a key regulator protein family, MAP65, as shown on Figure 1.

Phragmoplast is a plant cell specific structure essential for de novo cell wall formation [8]. The complete phragmoplast appears in parallel with the disassembly of mitotic spindle and is characteristic for telophase and cytokinesis. It contains microfilaments, antiparallel arrays of MT bundles, Golgi-derived vesicles bound to MTs and a wide set of macromolecules including microtubule associated proteins (MAPs) that regulate assembly and functioning of this peculiar structure. Golgi is the place for the synthesis and/or maturation of many hemicelluloses and pectins and structural proteins of the primary cell wall. These macromolecules are packaged to trans-Golgi vesicles that will be delivered through the phragmoplast microtubules to the site of cell plate assembly. At this site, vesicles fuse to form the new plasma membrane surfaces and deposit their material into the rudimentary cell wall that is the cell plate. This consists mainly of callose (synthetized by plasma membrane) and pectin, together with a set of structural proteins. Growth of the cell plate is centrifugal, starting in the center of the division site and expanding towards the pre-existing side walls. This phenomenon parallels the expansion of phragmoplast that is also of the centrifugal direction, disappearing at the central region where the cell plate is already formed [8]. The areas with still persisting phragmoplast are called the leading zone and sites with disappearing MT arrays are the lagging zone [13,14]; Figure 1. Much focus has been drawn on the regulatory roles of PP2A in the organization of the cortical microtubule (CMT) array and preprophase band (PPB). The related roles of B regulatory subunits and other regulatory proteins associated to PP2A/C-featuring TON2/FASS and TON1 are known [15,16]. *Arabidopsis* single mutations of A and C subunits of PP2A did not affect phragmoplast formation [17], however as we will see below, there is some evidence showing that events related to cell plate formation are regulated by this protein phosphatase. Inhibitors of PP2A (and the closely related phosphatase PP1) induce phragmoplast disruption and defects in cell plate formation [18,19,20].

MAP65 family is one of the most important factors that integrates MT stability and dynamics with vesicle delivery, fusion and, hence, cell plate formation in plants. MAP65 is a protein family belonging to a larger MAP family in eukaryotes with Ase1 in yeast and PRC1 in humans [21]. MAP65 comprises nine members in *Arabidopsis* [22,23]. These are middle and large sized proteins in the 54–80 kDa range [24,25,26]. Their N-terminal domains are thought to be responsible for MAP-65 dimerization and the C-terminal domains bind MTs. MAP-65 dimerization is important, since it assures MT cross-linking by this protein ([27,28] and Figure 1). Ser and Thr residues of their C-terminals are the sites of phosphoregulation [9,28]. Although there is a large amino acid sequence diversity of MAP65 family members, their C-terminal, that is, MT binding and phosphorylation–dephosphorylation domains, are highly conserved [23,26,29]. Based on a series of elegant experiments, Smertenko et al. [9] constructed a model for the phosphorylation dependent MT binding of *Arabidopsis* MAP65-1. MAP65 proteins are responsible for bundling and stabilization of plant MTs [9,22,30]. This activity is exerted at their dephosphorylation mainly by PP2A, while phosphorylation (see the MAPK actors below) has a contrary effect: debundling and destabilization (followed by depolymerization) of MTs [9,23,31]. Okadaic acid (OA), an inhibitor that decreases the activity of PP2A preferably over PP1, induces hyperphosphorylation of AtMAP65-1, causing excessive MT debundling [9,32]. PRC1, the mammalian counterpart of MAP65 is also regulated by PP2A and PP1 also catalyzed dephosphorylation at its MT binding C-terminal domain. These phosphatase activities will promote MT binding, thus bundling activity of PRC1 at the spindle midzone [21]. Later on it turned out that multiple members of the MAP65 family including MAP65-3 are targets for phosphorylation by phragmoplast-specific kinases and MAPKs [23,24]. MAP65 phosphorylation has been demonstrated for both *Arabidopsis* and tobacco [24,30]. The role of MAPK pathway(s) in the regulation of plant cytokinesis has been known for a relatively long time (see [33]). A MAPK cascade is involved in this, where MAPK4 (NRK1/NTF6 for tobacco) is the actor that phosphorylates MAP65. For *Arabidopsis* MAP65 proteins, phosphorylation at the C-terminal is catalyzed by CDK and MAPK6 as well and dephosphorylation (at least for MAP65-1) occurs at the same sites by PP2A [9,23,30,31]. If NRK1-nonphosphorylatable NtMAP65-1 is overexpressed, phragmoplast expansion will be delayed due to the inhibition of MT depolymerization at the phragmoplast lagging zone. If non-functional NRK1 is overexpressed, multinucleate cells with incomplete cell plates will form [30,34]. This is a very important finding, since at least in tobacco, it demonstrates the role of MAP65-1 dephosphorylation in cytokinesis, and as shown previously, this MAP is regulated by dephosphorylation via PP2A.

Besides MAPK cascades, the alpha-type Aurora kinases are involved in the regulation of phragmoplast formation and cell plate assembly. They phosphorylate MAP65-1 [35,36]. The phosphatase counterpart of Aurora kinases is PP2A in protein phosphoregulation of mitotic animal cells [17]. No such relationship has been demonstrated for plants.

MAP65-3/PLEIADE and MAP65-4 are the main MAP actors in plant cytokinesis. MAP65-3 has a bundling activity of antiparallel MTs at their + ends in the phragmoplast midzone ([22,28]; Figure 1). Thus, it has a key role in the organization of phragmoplast MTs [13]. Surprisingly after the experiments of Smertenko et al. [9] on MAP65-1 presented above, the role of protein phosphatases on the regulation of MAP65-3 binding to MTs was not studied much. As for other members of the MAP65 family, the C-terminal domain is responsible for MT binding [27] and hence, cross-linking and this activity is phosphorylation dependent. Mutant phenotypes of MAP65-3 are characterized by disrupted phragmoplasts and a cell plate that will result in the formation of multinucleate cells in *Arabidopsis* root tips. This will alter whole root development by disrupting normal patterning of histogenic apical meristem cells (see [37] for an example). The interaction of MAP65-3 with vesicle delivery and membrane fusion during cell plate biogenesis is discussed in the following sections. We can assume that PP2A has an important regulatory role in this (Figure 1). The regulation of MAP65 (major members of the family) phosphorylation state by PP2A may be exerted by two mechanisms: direct dephosphorylation of MAP65s and/or regulation (dephosphorylation) of MAPKs that phosphorylate MAP65 [38,39]. The above models are hypothetical, thus intense research is required in the field. We have two arguments in favor of MAP65 (including MAP65-3)-protein phosphatase interaction in this context. Firstly, NtMAP65-1 is involved in the timing of phragmoplast expansion, thus speed of cell plate formation and MAP65-1 homologues are known to be the subject of dephosphorylation by PP2A [9,30]. Secondly, C-terminal sites for MT binding and Ser/Thr phosphorylation–dephosphorylation of all MAP65 family members are conserved. PRC1, the mammalian counterpart of MAP65 is also a PP2A/PP1 target [21], indicating that phosphatase-mediated regulation is universal or widespread for these MAPs.

Dephosphorylation of MAP65 proteins may regulate indirectly vesicle delivery via the TRAPPII complex and vesicle fusion/cell plate development via regulation of KNOLLE–KEULE complexes, as summarized below.

#### 2.1.1. Vesicle Delivery through MTs and Their Release at the Phragmoplast.

Phosphoregulation at the ER–Golgi level of traffic for the vesicles containing cell wall materials is not known. During cytokinesis, MAP65-3/PLEIADE interacts with a protein complex called Transport Protein Particle II (TRAPPII) [13,28,31]. This complex consists of 10 subunits, is integrated into trans-Golgi network (TGN) derived vesicle membranes and has a membrane tethering function during cytokinesis. This tethering function contributes to vesicle recruitment at the site of cell plate formation [13,28]; Figure 1. In the absence of MAP65-3, TRAPPII shows a diffuse appearance in the cytosol at the end of mitosis [31]. It seems that MAP65-3 recognizes TRAPPII containing vesicles through the TRAPPII subunits TRS130/CLUB and TRS120 [28,31]. Phenotypes of TRAPPII mutants include fragmented cell plates [28]. When a mutant for MAP65-3 with a viable phenotype was doubled with a TRS120 mutant, the seedlings showed pronounced alterations in growth as compared to the single *trs120* mutant [31]. Phosphorylated MAP65-3 is equivalent to non-functional MAP, because it cannot bind to MTs (see the above subsections). According to available data, we could construct the following model (Figure 1). At the lagging zone of phragmoplast, MAP65-3 molecules are phosphorylated, and thus release MTs. MTs will depolymerize and release TRAPPII containing vesicles towards the growing cell plate. MAP65 is phosphorylated through a MAP kinase cascade. This is controlled by key cell cycle regulators. During telophase/cytokinesis, CDKB is downregulated, triggering a MAPK cascade that will phosphorylate MAP65-3 [31]. Since all members of the MAP-65 family are controlled by PP2A, we can predict that the phosphatase is not functional at this stage and site, therefore MAP65-3 remains phosphorylated and subsequently releases MTs at the lagging zone of phragmoplast (see more explanations at Figure 1). This hypothesis should be confirmed by further experiments.

#### 2.1.2. Vesicle Fusion during Cell Plate Formation

TRAPP II containing vesicles driven by dephosphorylated MAP65-3 are participating in membrane fusion at the cell plate assembly by interacting with the KEULE–KNOLLE system. MAP65-3 interacts with the SM protein KEULE [13]. SM proteins are clasp-like molecules that bind to t-SNAREs and facilitate their membrane-fusing activity in eukaryotes [40,41]. KEULE is cytosolic at the onset of mitosis, and it is recruited to phragmoplast MTs in telophase/cytokinesis. At the cell plate level, KEULE interacts with KNOLLE, a t-SNARE (Qa SNARE) crucial for membrane fusion during plant cytokinesis. As KEULE, KNOLLE is recruited to the division site via TGN derived vesicles [42]. KEULE is “clasping” KNOLLE, to maintain its fusogenic activity in the so-called trans-SNARE complexes, where proteins involved in membrane fusion are forcing membranes to “approach” each other, a key mechanism contributing to fusion [42,43,44]. *KEULE* mutants are impaired in membrane traffic, correct cell plate assembly and interestingly, in the turnover of phragmoplast MTs [42]. Several *KEULE* phenotypes resemble those of strong *MAP65-3/PLE* phenotypes that made the MAP65–KEULE interaction plausible. The current hypothesis is that the KEULE interaction with MTs stabilizes phragmoplast at the leading zone via MAP65-3 [42]. The KEULE-MAP65-3 interaction appears to be indirect and mediated through TRAPPII, since (i) KEULE localization to the cell plate was not impaired in *ple-4* mutants and (ii) MAP65-3 coimmunoprecipitates with the CLUB/AtTRS130 subunit of TRAPPII, but not with KEULE [31,42]. Overall, we can suppose that TRAPPII–KEULE interaction impedes the latter to regulate KNOLLE. Due to MAP65-3 phosphorylation, MTs are destabilized at the lagging zone of the phragmoplast. As a consequence, TRAPPII containing vesicles detach from depolymerizing MTs and will release KEULE that will be targeted to the cell plate membrane. The KEULE–KNOLLE interaction is switched on and vesicle fusion to the growing cell plate can occur (Figure 1). PP2A may act indirectly on this process by stabilizing MTs at the leading zone through dephosphorylation of MAP65-3 (see above). According to this model, the lack of phosphatase activity at the lagging zone will be one of the factors that release KEULE (see Figure 1 and previous paragraph on the possible interaction between TRAPPII and PP2A).

Overall, the comprehensive understanding of PP2A-mediated regulation of cytokinetic vesicle traffic and fusion during cell plate assembly are major future research directions.

### 2.2. Pathways of Autophagy in Relation to PP2A; Developmental Aspects

Autophagosomes are vesicular structures distinct to vacuoles formed during biotic/abiotic stress induced cell death or differentiation of certain cell types (elements of xylem or maturation of cereal aleurone cells). Their biogenesis starts from autophagophores that will engulf macromolecules or membrane compartments. In the next step they close, thus becoming double-membrane vesicles called autophagosomes. These vesicles expand, dock to the tonoplast of lytic vacuoles and fuse with them. Following this, their contents will be degraded inside the lytic vacuole [11,45]. The first requirement for autophagosome biogenesis is stress induced blocking of the TOR kinase activity of the Target of Rapamycin Complex 1 (TORC1). This blocking phosphorylates a set of proteins encoded by autophagic genes (ATG) [11,46]. The TORC-mediated pathway is required for the regulation of metabolism that promotes normal growth and development and its blocking is triggered by several unfavorable conditions, e.g., carbohydrate starvation [46,47]. The TOR-mediated signaling involves many (reversible) protein phosphorylation events, thus the involvement of protein phosphatases is obvious. PP2A is involved in the initiation of the autophagic pathway, as we will see in the sections below. However, not much is known for its effects on autophagosome fusion with the lytic vacuole.

**Figure 2:**
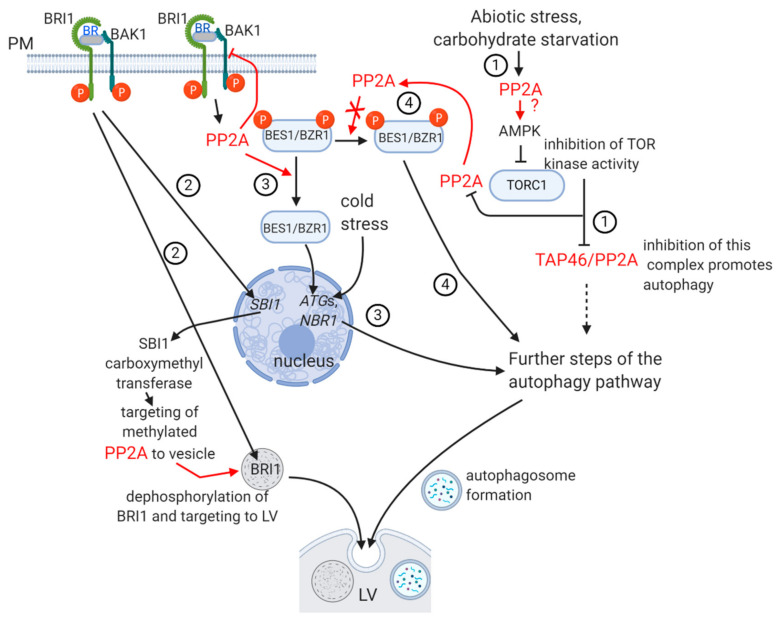
The regulation of autophagy and related brassinosteroid (BR) signaling pathways by PP2A. PP2A involvement is shown by red characters/arrows. TORC has various subcellular localizations in plants. We concentrate on autophagic pathways, therefore do not present here the whole signaling pathways mediated by blocking of TOR kinase signaling and by BR. Different pathways are indicated by numbers. Pathway 1: the autophagy pathway induced by inhibition of TOR kinase. The PP2A–AMPK relationship is still uncertain in plants, this is shown by a question mark. Pathway 2: induction of SBI1 methylase to remove excess BRI1. Pathway 3: induction of autophagy genes by activated BES1/BZR1, a result of BR signaling. Pathway 4: targeting of inactive BES1/BZR1 to the autophagy pathway. More details can be found in text. The upper/right part of the figure shows that during carbohydrate starvation, PP2A can be both activated and inhibited (Pathways 1 vs. 4). This may depend on the cell type dependent variation of (B and/or A) regulatory subunits. AMPK—AMP activated kinase; BR—brassinosteroid; BRI1 and BAK 1 is the BR receptor complex; LV—lytic vacuole, PM—plasma membrane. Figure was constructed with the aid of BioRender.com.

The protein phosphatase (PP2A and PP1) inhibitor microcystin-LR (MCY-LR) induces the fragmentation of large vacuoles into small tonoplast-coated vesicles in hypocotyl cells of young *Arabidopsis* seedlings. The formation of autophagosome-like structures is induced distinctly to vacuolar fragmentation [48]. Vacuolar fragmentation can be a rapid symptom of cell death [49]. Interestingly, knockout of the gene for PP2A-like yeast Sit4p blocks a signaling pathway regulated by vacuole associated TORC1. This stops vacuole fission-mediated lysosome formation [50]. In yeast, PP2A is controlled by a phosphatase regulatory subunit, Tap42, phosphorylated by TOR-mediated signaling. This will lead to the formation of Tap42-PP2Ac complexes, and phosphatase activity will be activated or inhibited by changing the substrate specificity of PP2A. Blocking of TORC-mediated signaling inhibits PP2A in yeast. The consequence is the inhibition of fission-mediated lysosome, but stimulation of autophagosome formation [51,52]. In *Arabidopsis*, TOR phosphorylates Tap46, as for yeast Tap42. Triggering of PP2A activity by phosphorylated Tap46 in plants will inhibit the formation of the characteristic double-membraned autophagosomes as for Tap42 in yeast ([4,51]; **pathway 1** of Figure 2). However, the effect of Tap46 on PP2A activities is largely contextual (as Tap42-PP2A interaction in yeast), for example, it inhibits PP2A during stress-related ABA signaling [53]. The biogenesis and structure of autophagosomes appears to be distinct to those of provacuolar compartments (PVCs) and vacuoles [54]. However, as shown above, inhibition of protein phosphatases by MCY-LR in plants and knockout or misregulation of PP2A in yeast affects both vacuole organization and autophagosome biogenesis, presumably via partially common pathways.

Sequestration of autophagic membranes, presumably from the ER, is regulated by phosphatases in mammals [55]. The autophagy pathway involves the regulation of serine-threonine kinases at several steps, and this is valid for plants, too. AMP-activated Ser kinase (AMPK) blocks the Ser-Thr kinase activity of TORC1. In eukaryotes other than plants, AMPK is regulated by PP2A, while in plants, it seems that both PP2A and PP2C play a role in this ([46,56]; pathway 1 of Figure 2). This event will trigger the activity of ATG proteins, featuring the ATG1/ATG13 complex and ATG8, which will lead to the formation of autophagosomes [57,58,59,60]. However, practically nothing is known on the direct involvement of PP2A in the ATG dependent pathways.

Brassinosteroid (BR) signaling interferes with autophagy in multiple ways, and as we will see, several of these are related to pathways mediated by the inhibition of TOR kinase activity (Figure 2):

When BR binds to its plasma membrane receptor, BRI1 (BRASSINOSTEROID INSENSITIVE1), it will associate with BAK1 and the transphosphorylation reactions between these two membrane proteins will trigger the BR-mediated signaling pathway [61]. In the absence of BR, BRI1 is recycled between plasma membrane and the cytosol via endosome vesicles [62]. **Pathway 2** of Figure 2 shows that in the presence of excess BR, a significant fraction of BRI1 is degraded in vacuoles in a PP2A dependent way. BR signaling induces the expression of SBI1, a leucine carboxymethyltransferase that methylates PP2A, targeting phosphatase to the endosomes involved in BRI1 recycling. BRI1 will be dephosphorylated, thus targeted to lytic vacuoles [4,62]. This endosomal traffic mechanism is very important for development. The *rcn1* (roots curl in naphthylphtalamic acid) mutants of *Arabidopsis* lack a functional “A” regulatory subunit of PP2A. As a consequence, BRI1 will be accumulated and BR signaling overstimulated [60]. This is one of the events leading to abnormal root development and gravitropic response of this mutant (see Section 2.3 as well).

#### Pathway 3 of Figure 2

Activation of BRI1 by BR leads to PP2A-catalyzed dephosphorylation of BES1/BZR1 (BRI1/EMS SUPPRESSOR1/BRASSINAZOLE RESISTANT 1) transcription factors that accumulate in the nucleus and control the expression of diverse (both growth and stress related) genes. PP2A is then inactivating BAK1, thus BR signaling is terminated by a negative feedback mechanism [4,58,61]. Cold treatment induces BZR1-mediated activation of autophagy genes *ATG2* and *ATG6*, and *NBR*s. The latter are selective autophagy receptors that recognize ubiquitinated protein aggregates that are formed during abiotic stress. NBR1 interacts with ATG8. Both ATGs and NBRs are involved in autophagosome biogenesis [63]. Similar induction of autophagy was observed, when BZR1 was overexpressed in tomato [64].

What is then the relationship between TORC-BR signaling-PP2A in relation to autophagy? This is summarized on Figure 2. Dark-induced carbohydrate starvation induced blocking of TORC signaling and led to BZR1 degradation via autophagy in *Arabidopsis*. This occurred probably by preventing BZR1 to be dephosphorylated by PP2A ([65]; **pathway 4** of Figure 2). Blocking of TOR kinase occurred probably via a BR-independent mechanism but induced the usual autophagy pathway. Inhibition of autophagy stabilized BZR1 levels. BR signaling inactivates BIN2 kinase by ubiquitination and subsequent degradation. Blocking of this signaling pathway by drought and starvation stress renders BIN2 active. This kinase will phosphorylate DSK2, an ubiquitin receptor that will bind ATG8. The DSK2–ATG8 complex will target BES1 for degradation [58,66].

To summarize the autophagy–vesicle traffic PP2A relationship: currently available data show that PP2A interferes with early steps of the autophagic pathways. We have no evidence for its direct role in autophagosome formation itself. PP2A is also regulating BR perception by targeting excess BRI to lytic vacuoles via vesicle traffic. Thus, it controls BR dependent plant developmental events.

Several important questions/future research directions regarding PP2A-mediated regulation of autophagy:

(i) What is the detailed mechanism of blocking of TORC-mediated signaling by PP2A (see Figure 2 as well)?
(ii) Are biogenesis and fusion of autophagosomes to the lytic vacuole regulated by PP2A?
(iii) Further evidence is needed to demonstrate that PP2A-mediated dephosphorylation of BZR, an important actor in BR signaling, is blocked during autophagy.

Interestingly, glucose induced deviation of *Arabidopsis* root vertical growth is enhanced by BR and this enhancement is significantly stronger in the *rcn1* mutants [67]. This mutant shows altered auxin efflux, because it influences intracellular traffic of PINs. In the next section we will discuss the interference of PP2A with traffic and recycling of PIN auxin efflux carriers, of major importance in plant development.

### 2.3. The Subcellular Fate of PINs in Relation to PP2A

Plasma membrane localization and recycling of PIN proteins by endomembranes are nice examples for the collaboration of membrane structures in integrated cell functioning. These events are regulated by reversible phosphorylation. The role of PP2A is well documented with many excellent papers in the field. This is true especially for PIN1 and PIN2. We will show a short overview of PP2A-mediated PIN vesicle traffic. We will also highlight several still not completely solved or little known aspects/questions and possible future research directions.

**Figure 3:**
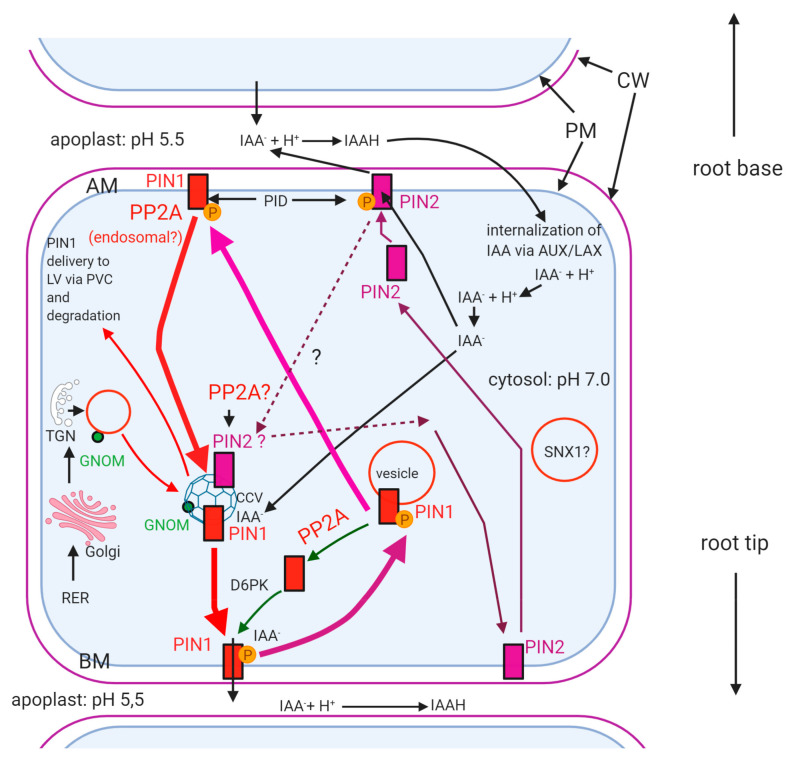
The role of PP2A in functioning, recycling and transcytosis of PINs. Although in general, PIN1 and PIN2 are working in different cells, here we show them in a single cell compartment to show their differential transport and functioning. For simplicity, only vesicle traffic pathways directly or indirectly dependent on PP2A are shown. For example, other GEFs than GNOM and Rab GTPases involved in the traffic of PINs are not shown here. In root epidermal cells, the site of auxin efflux mediated by PIN2 is opposite to efflux mediated by PIN1 in the stele. Pathways of PIN1 and PIN2 polarity shift are partially interfering. GNOM is presumably participating in the transcytosis of PIN1 and PIN2 as well, but for PIN2, this happens mainly in root cortex cells where it directs auxin transport towards the root apex. GNOM pathway-mediated transport of PIN2 towards basal membrane for recycling purposes is probable (dashed purple arrows) but needs further evidence. Both PIN-mediated auxin efflux and their vesicular transport are dependent on protein phosphorylation. PINOID kinase (PID) phosphorylates both PIN1 and PIN2 at the apical membrane, while D6PK kinase phosphorylates PIN1 in the basal pole of a root cell. PP2A dephosphorylates PIN1 and presumably PIN2 at the apical pole and PIN1 at the basal pole. Question marks show yet not fully investigated events. At the basal membrane, PIN2 might be phosphorylated by a yet unknown kinase and if it remains in a phosphorylated state here, its recycling will be impeded. **→** (Red arrow) GNOM dependent transcytosis of PIN1 in the apical-basal direction. **→** (Pink arrow) Basal–apical shift of PIN1 is independent of GNOM. **→** (Purple arrow) The pathway of intracellular PIN2 traffic. Thick arrows highlight the transcytosis and recycling pathway of PIN1. AM—apical membrane; BM—basal membrane; LV—lytic vacuole; PVC—prevacuolar compartment (multivesicular body/late endosome); SNX1—SORTING NEXIN1 containing vesicle; the figure was constructed with the aid of BioRender.com.

The role of protein phosphorylation in auxin polar transport with the involvement of PIN proteins is known for a relatively long time (see [68] for an early review). To date, eight members of the PIN (PIN-formed) family are known [12,69]. They are auxin efflux carriers and the higher MW members PIN1-4 and PIN7, with 10 transmembrane domains are the most important in regulating polar auxin transport, thus the proper tissue distribution of this crucial plant growth regulator [12,69]. Auxins (including indole-3-acetic acid/IAA, the most abundant one) and their distribution in the plant body are essential in nearly all aspects of plant development including cell cycle regulation, embryo and vegetative organ development, control of tropisms, etc. ([70,71] and many others). They are internalized mainly by the AUX1/LAX permease and externalized by PINs at the opposite plasma membrane surface ([12,72] and Figure 3). Functioning, recycling and degradation of PINs is the most studied for PIN1, PIN2 and PIN3). The direction of auxin flow in axial organs is mediated differently by these PINs. For roots, PIN1 (and to a lesser degree, PIN3 and PIN7) is transporting IAA from the base towards root tip through the stele, PIN2 is directing transport from the tip towards the base in the root cortex and epidermis—although it can also direct root tip directed transport in cortex cells—and PIN3, PIN4 and PIN7 function as “sorters” of IAA in the root cap columella [72,73,74]. PIN3, PIN7 and especially PIN2 are important for gravitropic responses [12,69,70]. Due to this differential orientation of auxins, it is likely that there are differences in the regulation of subcellular localization of different PINs. Inside the cell, deprotonated IAA is taken up by endomembrane localized PINs, then IAA is externalized. PP2A dephosphorylates PINs, which are then recycled by vesicular transport through the cytosol and ready to take up the next IAA^-^ ion. Thus, PP2A has a crucial role in regulating polar localization and functioning of PINs that control auxin distribution in the plant [75]. It is worth mentioning, that TOPP4, a PP1 isoform, is contributing to PIN1 dephosphorylation [76]. Recycling and PIN transcytosis to the site of auxin efflux is governed by the GNOM proteins, at least for PIN1 and PIN3 (see [70,77] for examples; Figure 3). GNOM proteins are GTP-GDP exchange factors (ARF-GEFs) for small G proteins that are involved in vesicle budding from diverse membrane structures. *gnom* mutants are characterized by inhibition of lateral root formation and dwarfism [78,79]. Transcytosis to the site of IAA^-^ efflux governed by GNOM containing clathrin coated vesicles (CCVs) occurs only when PIN1 is dephosphorylated by PP2A [80]. The GNOM family includes isoforms involved in lateral root development, regulation of developmental polarity and many other events [79]. GNOMs are important in the biogenesis of PIN1-transporting vesicles via the TGN [80]. Transcytosis, thus recycling of PIN1 and PIN2 to the opposite side—the site of uptake of IAA released by AUX1/LAX occurs only when PINs (at least PIN1, PIN2, PIN3 and PIN4) are phosphorylated by the serine-threonine kinase PINOID (PID) or other kinases (see below), via a GNOM independent pathway [12,70,81,82]. GNOM is also involved in the proper localization of PIN2 in root cortical cells and PIN3 during gravistimulation [83,84]. At this stage, the further fate of PINs is (i) either relocation to the plasma membrane via TGN and binding a new deprotonated IAA^−^ ion and transferring it to the efflux site or (ii) at least for PIN1 and PIN2, poly-ubiquitination and entering into a prevacuolar compartment (PVC/MVB) with a GNOM-independent mechanism, for delivery into the lytic vacuole and degradation ([68,75,83] and references therein; and Figure 3). Vesicular transport through PIN transcytosis occurs via microfilaments [80]. The way of PIN functioning and recycling made some researchers to find analogies between the mechanism of auxin efflux and neurotransmission [85].

For a more detailed view of PIN phosphoregulation: the “long” PINs have transmembrane domains and a large hydrophilic loop in the cytosol. This loop is the subject of reversible phosphorylation and it has multiple phosphorylation sites catalyzed by multiple kinases [75]. PINOID kinase (PID, a serine-threonine kinase) and the serine-threonine protein phosphatase PP2A are the two most well known antagonistic partners for the regulation of intracellular PIN localization by acting at the same sites of the hydrophilic loop of PINs [78,81]. PID mutants show very similar phenotype to *pin1*, with a pin-like shoot apex and low flowering rate, while mutants in the A regulatory subunit of PP2A alter auxin distribution and hence, induce root tip meristem collapse, similarly to PID overexpressing *Arabidopsis* plants [75]. PID can phosphorylate all the higher MW PINs [12,86]. It is a kinase belonging to the AGC (cAMP-dependent protein kinase A/cGMP-dependent protein kinase G/protein kinase C) family [83]. Other kinases involved in PIN phosphorylation and PIN localization/auxin transport are the D6 protein kinase (D6PK) and WAG1 and WAG2. They all belong to the AGCVIII kinase subfamily [12,75]. All these kinases are thought to have PP2A as the counteracting partner (see citations in [75]). The phosphatase counterparts are the C3 and C4 isoforms of PP2A catalytic subunit that were shown to be the key players in PIN dephosphorylation [87]. Members of two other protein kinase families, CRK5 and diverse MAP kinases, are also involved in PIN phosphorylation [12,88,89].

The many papers on PIN cellular-tissue distribution indicate that as for cell plate formation and autophagy pathways, PIN recycling and transcytosis seems not to be regulated by PP2A at the ER–Golgi vesicle delivery level. The protein phosphatase is rather influencing PIN traffic at downstream levels.

In the following sections, we show several not completely solved questions regarding phosphorylation/dephosphorylation related vesicular traffic of PIN and in general, auxin transport:

(i) Is internalization of IAA by AUX1/LAX regulated by reversible protein phosphorylation? This aspect is largely unknown.
(ii) What mechanism directs differential membrane localization of PIN1 vs. PIN2 directed auxin efflux? How reversible PIN phosphorylation is involved in this (see Figure 3)? As we could see above, the reversible phosphorylation of PINs regulates their plasma membrane polar localization. Dephosphorylation will trigger internalization and then recycling, usually to the opposite cell side [90,91]. Phosphorylation of PIN1, 2 and 4 by PID results in their localization in the apical membrane of root cells, while dephosphorylation will redirect them to the basal plasma membrane ([92] and Figure 3). If PIN1 is maintained in a phosphorylated state at the apical membrane, auxin will be transported towards root base and the apical meristem of main roots will be depleted of auxin. At this site, it is phosphorylated by PID—the function of this is stabilization of PIN1 ([75]; Figure 3). What is the reason for the lack of auxin efflux by phospho-PIN1 at the apical membrane under normal conditions? PIN1 phosphorylation at the basal membrane is needed for proper auxin efflux, at the basal poles of plasma membranes in roots [78,91,92,93,94]. For PIN2, the mode and effects of reversible phosphorylation are contrasting to PIN1. How is phosphorylation directed differentially in different cell types and for different PINs? Normally, PIN2 should be localized apically in cell membranes, to direct upward auxin flux towards root base mainly in root epidermal cells. If PIN2 remains hyperphosphorylated, it will remain in the apical membranes and blocks its recycling [75]. As PIN1, PIN2 drives auxin efflux, when it is phosphorylated. At the basal (facing the root tip) pole of the cell, PIN1 is phosphorylated by brefeldin A (BFA) sensitive D6PK to drive auxin efflux and its local recycling and PP2A dephosphorylates it to drive its transcytosis into the apical (shootward) cell pole. In contrast to PIN1, PIN2 is not phosphorylated by D6PK at the basal pole of the cell, because its hydrophilic loop lacks the phosphorylation sites for D6PK [75]. Recently it has been shown that PID has a non-polar distribution, that is, it can be localized practically at any membrane sites [12]. Why then, its activity is “polarized” for PINs?

PIN1At, a peptidyl-prolyl cis/trans isomerase changes the conformation of PIN1 at the serine 337 site phosphorylable probably by other kinases than PID. This event contributes to the stabilization of phospho-PIN1 at apical membranes that will be relocated to the basal root cell membrane by PP2A. Interestingly, PIN1At is not interacting with other PINs than PIN1 [95]. Therefore, it was proposed that the phosphoregulation of PIN2, 3 and 7 is performed at least partially by different mechanisms to PIN1 [12]. Corresponding to this, we propose that phosphatases exert post-translational modifications on different PINs at different membrane locations, by different regulatory mechanisms, but we still do not understand this process. Further exciting research is expected in this field.

Friml et al. [92] further developed a hypothesis drawn by Sachs [96]. He proposed that the polarity shift of PINs is driven by auxin itself. Moreover, membrane localization of different PINs is changed differentially by auxin in different cells by a TIR dependent pathway ([80] and references therein). A challenging question is whether IAA can drive the phosphorylation/dephosphorylation of PINs, thereby regulating the membrane site of auxin efflux. Is it possible that membrane-level PP2A activities are triggered by auxin?

(iii) Which A/B regulatory subunit(s) of PP2A are responsible for regulating phosphatase activity, correct targeting and substrate specificity to PIN? It was shown that the A scaffolding subunit of PP2A is not only stabilizing the holoenzyme, but it has other important regulatory functions [12]. RCN1 is an A (Aα) regulatory subunit of PP2A, important in polar auxin transport, thereby regulating mitotic activity in root apical meristem, root morphology, hypocotyl apical hook formation and axial organ elongation. PP2A/A mutations are altering root development and gravitropism by changing auxin transport, thus distribution [12,97]. In the *rcn1* (roots curl in naphthylphtalamic acid) *Arabidopsis* mutant, PP2A activity was reduced, indicating this subunit is important for maintaining functionality of the holoenzyme. The loss-of-function of specific PP2A/A subunits results in the basal-to-apical shift of PIN1 membrane localization [68,70,93]. The A1 subunit is binding to phosphatidic acid derived from the activity of phospholipase D (PLD), thus it is crucial for the PIN1-related membrane localization of PP2A. Inhibition of PLD and/or mutated PP2AA1 results in the inhibition of PIN1 recycling leading to its mislocalization, i.e., accumulation on apical membranes [98]. However, it is still not clear why this mechanism is restricted to specific membrane areas? Is PLD localization polarized to drive polar localization of PIN1?

Another PP2A regulatory subunit found to influence PIN dephosphorylation is ROTUNDA3 [99]. The loss-of-function mutant phenotype shows root and shoot developmental defects associated with altered auxin distribution (accumulation in shoot tip and depletion in root tip). The nature of this regulatory subunit is to be characterized by further research.

As we could see, good knowledge is available on PP2A/A subunits in relation to PIN. The main question is: how B regulatory subunits of PP2A affect functioning and vesicle traffic of PINs? As we have mentioned before (see the Introduction section), these subunits are important in substrate specificity, subcellular localization and regulation of enzyme activity of the PP2A holoenzyme. The *fass* mutations of *Arabidopsis* affected in a B’’ regulatory subunit are dwarf and show abnormal radial expansion of cells both in roots and hypocotyls. FASS (TON2) is part of a complex that targets PP2A to MTs [17]. We still do not understand well whether this subunit of PP2A affects vesicle transport/membrane localization of PINs and/or auxin transport.

(iv) Why only dephosphorylated PIN1 (and PIN2) is recycled via the GNOM pathway? There is evidence that phosphorylated PINs are not preferred by the GNOM-mediated vesicle traffic pathways. For example, *Arabidopsis pp*2*aa1* plants are hypersensitive to BFA and GNOM-mediated transcytosis of PIN2 is impaired, probably because PIN2 remains phosphorylated due to the immobilizing of PP2AA1, thus preventing proper PP2A activity at the correct location. [77,84]. It was shown that GNOM decreases the abundance of PINOID, thus the level of phosphorylated PINs [84]. Is this inducing a conformation change of PINs that will facilitate GNOM-mediated vesicular transport? We are still far away from understanding the mechanism related to the above question.
(v) Why only dephosphorylated excess PIN is poly-ubiquitinated and delivered to lytic vacuoles for degradation? PIN1 and PIN2 are targeted to the vacuole via other ARF-GEFs than GNOM [74]. How its PP2A-mediated dephosphorylation governs this pathway? What is the signaling mechanism involved?

## 3. Concluding Remarks

In this review we show that regulation of plant vesicle traffic by the serine-threonine protein phosphatase PP2A is involved in diverse processes crucial in plant development and stress responses. To date, there is no evidence for phosphatase-mediated regulation of endo and transcytotic events directly at the ER–Golgi level in the plant cell. Downstream events of vesicle traffic, on the other hand, are regulated, directly or indirectly, by PP2A. Target proteins as regulated by PP2A are shown on Table 1. Vesicle delivery is strongly dependent on the cytoskeleton. MAP65-mediated MT stability and bundling depend on reversible protein phosphorylation. This is of crucial importance in the regulation of vesicle delivery during cytokinetic de novo cell wall formation. PP2A does regulate directly vesicle delivery via phragmoplast MTs: by dephosphorylating MAP65-3, this MAP will be able to bind MTs, maintaining their stability. Another important event is autophagy occurring during responses to environmental stresses and cell death. Current evidence suggests that diverse stresses (unfavorable environmental conditions, starvation) and PCD involving differentiation of specific plant cell types activate particular PP2A holoenzyme complexes that block TORC-mediated signaling, thereby trigger the autophagy pathway. Interestingly this mechanism has common points with stress related BR signaling. PP2A-mediated regulation of vesicle-driven recycling of the BR receptor, BRI, contributes essentially to tissue differentiation/whole-plant development. Since BR may influence auxin transport and signaling, it is possible that PIN-mediated auxin efflux and the intracellular vesicle traffic of PINs are not completely independent to BR signaling. Phosphatase-mediated vesicular delivery and recycling of PINs are well described, although there are many unsolved questions in this field. In general, there are plenty of exciting future research directions related to the PP2A regulated vesicle traffic and its developmental consequences. Here are the most important directions:

- How PP2A regulates vesicle fusion at the formation of cell plate? Is maintenance of phosphorylated state of MAP65 at the division plane caused by local PP2A inhibition? Does downregulation of PP2A at this level influence directly cell patterning in meristems?
- Is PP2A regulating directly the biogenesis of autophagosomes and their fusion to the lytic vacuole?
- How PP2A regulates differential vesicle traffic, membrane localization and functioning of different PINs? This research will contribute to a better understanding of auxin-regulated development of axial organs.

## Figures and Tables

**Table 1:** Examples for the involvement of PP2A (PP1) in vesicle traffic of plant cells. See text for more details.

Subunit Involved	Change of Protein Phosphatase Activity or Other PP2A Related Functions	Target Protein	Function	References
PP2A/C	activation	MAP65-1 MAP65-3?	Bundling of MTs, indirect regulation of vesicle traffic	[9,30]
PP2A/C	activation	AMPK, activation	Blocking of TORC pathway, triggering of autophagy	[56]
TAP46 subunit of PP2A	activation	proteins of the TORC pathway	Triggering of the TORC pathway and blocking of autophagy pathway	[4,51]
Methylation of PP2A/C by SBI1	SBI1 targets PP2A to BRI1 containing endosomes	BRI1	Dephosphorylation of excess BRI1 that will be targeted to lytic vacuoles	[4,62]
PP2A/B’, C	inhibition/deactivation	BZR1	BZR1 remains phosphorylated, thus targeted to the autophagy pathway	[65,100]
PP2A/A, C3, C4, PP1	activation	PINs, dephosphorylation	PIN recycling and proper membrane localization. Polar transport-tissue distribution of auxins	[69,70,76,87,98]

AMPK—AMP-activated Ser kinase; BRI1—brassinosteroid (BR) receptor; BZR1—BR induced transcription factor; SBI1—leucine carboxymethylase.

## Data Availability

Not applicable.
